# Two-Dimensional Restructuring of Cu_2_O Can Improve the Performance of Nanosized n-TiO_2_/p-Cu_2_O Photoelectrodes under UV–Visible Light

**DOI:** 10.1021/acsami.1c13399

**Published:** 2021-10-04

**Authors:** Antonio Rubino, Robertino Zanoni, Pier G. Schiavi, Alessandro Latini, Francesca Pagnanelli

**Affiliations:** Department of Chemistry, Sapienza University of Rome, P. le Aldo Moro 5, 00185 Rome, Italy

**Keywords:** electrodeposition, nanosized p−n heterojunctions, n-TiO_2_ nanotubes, photoelectrochemical cells, photoinduced restructuring, 2D leaf-like p-Cu_2_O

## Abstract

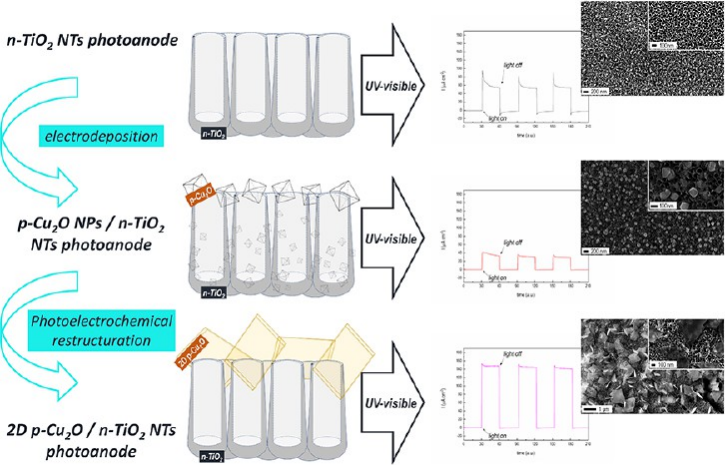

p-Cu_2_O/n-TiO_2_ photoanodes were produced by electrodeposition of octahedral p-type Cu_2_O nanoparticles over n-type TiO_2_ nanotubes. The photoresponse of the composite p–n photoanodes was evaluated in photoelectrochemical cells operating at “zero-bias” conditions under either visible or UV–vis irradiation. In both operating conditions, the produced electrodes invariably followed the p–n-based photoanode operations but exhibited lower photoelectrochemical performance as compared to the bare n-TiO_2_ photoanode under UV–vis light. The reported experimental analysis evidenced that such decreased photoactivity is mainly induced by the scarce efficiency of the nanosized p–n interfaces upon irradiation. To overcome such limitation, a restructuring of the originally electrodeposited p-Cu_2_O was promoted, following a photoelectrochemical post-treatment strategy. p-Cu_2_O, restructured in a 2D leaf-like morphology, allowed reaching an improved photoelectrochemical performance for the p–n-based photoanode under UV–vis light. As compared to the bare n-TiO_2_ behavior, such improvement consisted of photoanodic currents up to three times larger. An analysis of the mechanisms driving the transition from compact (∼100 nm) octahedral p-Cu_2_O to wider (∼1 μm) 2D leaf-like structures was performed, which highlighted the pivotal role played by the irradiated n-TiO_2_ NTs.

## Introduction

Over the past two decades, increasing energy demand accompanied by the need to decrease both carbon dioxide emissions and the dependence on fossil fuels^1^ has accelerated the development and optimization of technologies useful to generate electric energy from renewable sources, including solar, wind, and geothermal.^2^ However, the application of these technologies is currently limited by the characteristic intermittence of renewables,^3^ causing one to turn to electric energy storage technologies, including batteries and accumulators, which currently cannot guarantee the required autonomy in many applications.

An alternative strategy is to store renewables as chemical feedstock,^4^ as for hydrogen production by photoelectrochemical (PEC) water splitting.^5,6^ PEC devices, thanks to the employment of semiconductors as electrode materials, exploit the absorbed light energy as the “power supply”, driving the electrochemical reactions within the cell.

Specifically, in PEC cells (Supporting Information, Figure S1), n-type and p-type semiconductors act, respectively, as photoanodes (catalyzing water oxidation) and photocathodes (catalyzing water reduction).^6−8^

In this framework, TiO_2_ has emerged as one of the most investigated n-type semiconductors.^9−11^ Considerable attention has been devoted to the application of TiO_2_ in the form of a high-aspect-ratio nanotubes array^12,13^ to produce photoelectrodes for PEC cells.^14,15^ In fact, nanotubes (NTs) can be easily generated and tailored in length and diameter by the potentiostatic anodization of titanium substrates.^13,16,17^ However, TiO_2_ is characterized by a wide band gap (∼3.2 eV for anatase), which restricts the light absorption to wavelengths in the UV, a small percentage (∼5%) of the available incident light.^18^ The doping of TiO_2_ has been proposed,^19,20^ which, however, produced a slight widening of the useful wavelengths in the visible region. A wider visible range can be effectively exploited by forming a p–n heterojunction through the coupling of n-TiO_2_ with a p-type semiconductor characterized by a sensibly narrower band gap.^21,22^ The resulting system can be employed as the photoanode in PEC devices (Supporting Information, Figure S1(II)). In this framework, considerable efforts have been devoted to optimization of the photoelectrochemical performance of n-TiO_2_/p-Cu_2_O heterojunction systems.^23−25^

Cuprous oxide is characterized by a quite narrow band gap (2.0–2.6 eV), which makes it suitable for substantial visible light absorption.^26−28^ However, in nanosized p–n systems, efficient light absorption can be guaranteed only by tightly controlling the size and morphology of the Cu_2_O nanoparticles (NPs) deposited over the TiO_2_ NTs array. As reported by Musselman et al. about the role of the p-Cu_2_O in a bilayer-based p–n heterojunction system, the Cu_2_O thickness should increase from a few hundred nanometers in the UV region to several micrometers in the visible to have efficient light harvesting.^29^ It could be concluded that to induce an increased photoresponse of a n-TiO_2_/p-Cu_2_O heterojunction under UV–vis, it suffices to increase the Cu_2_O sizes. On the other hand, as the same authors indicate, this can cause a poor charge collection efficiency at the p–n interface, limiting the useful device thickness.^29^ In fact, since the Δ*V*_photo_ responsible for the p–n-based photoanode operations is determined by the space-charge layer formed at the p–n interface, p-Cu_2_O dimensions larger than the width of the space-charge layer can result in photogenerated charge carriers which recombine in the p-side instead of contributing to Δ*V*_photo_ (Supporting Information, Figure S2).^29^

Electrodeposition is a cost-effective method to control the size and morphology of the p-Cu_2_O on n-TiO_2_,^30,31^ by which a large range of particle sizes and morphologies can be easily obtained.^30,32−34^ Accordingly, numerous studies have investigated the photoelectrochemical performance of p-Cu_2_O electrodeposited onto n-TiO_2_.^24,30,34−37^ The focus of such studies was on the application of TiO_2_ NTs with Cu_2_O nanostructures, which were almost invariably electrodeposited from an alkaline copper ion solution in lactic acid.^30,34,37^ Most of these studies concluded that Cu_2_O electrodeposition can improve the photoelectrochemical performance, as compared to the bare TiO_2_ NTs. However, closer analysis of the published data may raise some doubts about these conclusions. In fact, the basis for claiming an improved photoelectrochemical performance was frequently the observation that the synthesized electrodes produced a non-negligible photoresponse under visible light.^38−40^ Two considerations can be drawn: (i) bare n-TiO_2_ inactivity has been tested under only visible irradiation (λ > 400 nm); (ii) in nanosized heterojunctions employing p-Cu_2_O, cutting the UV light component unavoidably reduces the overall light-harvesting efficiency.^29^ Therefore, a comparison of the bare n-TiO_2_ and composite n-TiO_2_/p-Cu_2_O electrode performance under simultaneous UV–vis irradiation is definitively required to quantify the real impact of p-Cu_2_O electrodeposition. In fact, as reported by Tsui et al.,^41,42^ the electrodeposition of Cu_2_O NPs over the TiO_2_ NTs array produced, as the main effect, a strong decrease in the UV light-harvesting efficiency, from about 10% to 1%, accompanied by a very slight increase, from 10^–3^% to 0.2%, induced over the visible region (λ > 400 nm). In this scenario, very few papers have reported improved performance under UV–vis irradiation.^39,43,44^ In the latter cases, it is not fully clarified if the improved performance is exclusively attributable to the role of the electrodeposited Cu_2_O rather than to the application of a direct bias during the photocurrent tests performed.^39,43^ As an example, in the work by Bai et al., photocurrent tests under UV–vis irradiation are carried out at a 0.5 V (vs Ag/AgCl) applied bias, and the photoelectrocatalytic activity is greatly enhanced as compared to photocatalysis (i.e., without bias application).^44^ In the same work, the performance of the composite TiO_2_/Cu_2_O electrodes increases with increasing Cu_2_O content up to a maximum and eventually decreases when the Cu_2_O content is doubled,^44^ suggesting that the Cu_2_O size also influences the overall composite electrode performance, even upon direct bias application. This result is in agreement with our previous findings, where we reported that the photocatalytic activity of the composite TiO_2_/Cu_2_O in the absence of bias progressively deteriorates at increasing Cu_2_O loading,^45^ which corresponds to an increase in the sizes of the electrodeposited Cu_2_O.^46^

In contrast, p-Cu_2_O electrodeposition significantly improves the photoelectrochemical performance at zero-bias conditions of p–n systems based on n-TiO_2_ substrates other than NTs.^24,33,37,47^ Specifically, more efficient p–n photoanodes were produced by replacing NTs (∼10 nm wall thickness) with nanosheets (∼400 nm thickness)^24,33^ or a continuous planar layer.^47^ However, the increased efficiency induced by the thicker TiO_2_ substrates (∼60–100 μA/cm^2^)^24^ was outweighed by losses determined by the reduced surface/volume ratio (∼50–200 μA/cm^2^ for the bare TiO_2_ NTs characterized by ∼0.5–2 μm tube length and ∼100 nm inner diameter).^44,48^

The present investigation illustrates the advantages of a particular modification operating on the morphology and size of the Cu_2_O nanostructures and explores the mechanisms driving the morphology transitions of the Cu_2_O deposits. The photoresponse of such nanosized p–n system was evaluated by testing the obtained photoelectrodes in a PEC cell operating at zero-bias conditions under pulsed UV–vis irradiation.

## Results and Discussion

### Photoelectrochemical Performance of the n-TiO_2_/p-Cu_2_O-Based Photoanodes

**Figure 1:**
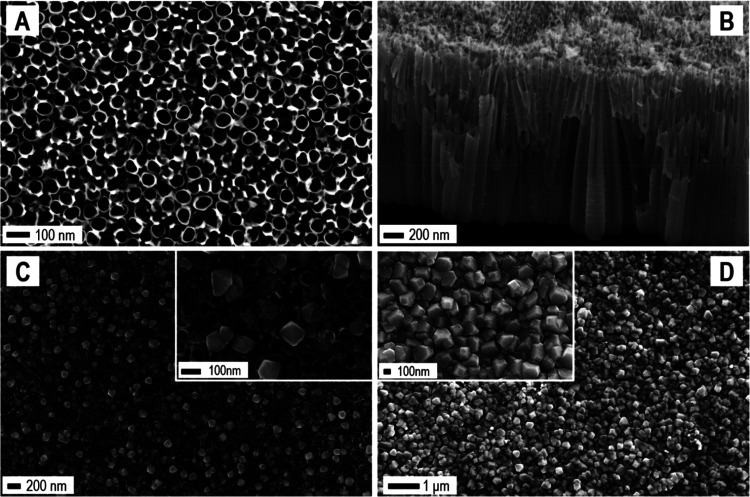
FE-SEM images. Bare TiO_2_-based electrode: top view (A) and cross-sectional view (B). Top views of the TiO_2_/Cu_2_O electrodes obtained at different Cu_2_O loadings: 120 (C) and 500 mC (D) transferred charge, with the related insets, taken at higher levels of magnification.

Figure 1 shows the FE-SEM images of the bare n-TiO_2_ NTs electrode produced by titanium anodization (Figure 1A and 1B) and of the composite n-TiO_2_/p-Cu_2_O produced by successive Cu_2_O electrodeposition on n-TiO_2_ NTs at 120 (Figure 1C) and 500 mC transferred charge (Figure 1D). Under the electrodeposition conditions tested, Cu_2_O deposits exhibited a predominant octahedral morphology (Figure 1C),^45,46^ which progressively led to a continuous polyhedral layer, upon increasing the transferred charge to 500 mC (Figure 1D). A cross-sectional view of the composite electrode at 120 mC is shown in the Supporting Information (Figure S3), highlighting the reduced sizes of the Cu_2_O NPs along the NTs walls as compared to the top surface of the NTs array.

Characterization by XRD (Supporting Information, Figure S5) confirmed the effectiveness of the implemented electrodeposition method in inducing the formation of Cu_2_O crystal phases at room temperature (Figure S5c and S5d) according to our previous findings.^46^ Elemental analysis by EDX on the sample electrodeposited at 500 mC, synthesized ad hoc to minimize the contribution of the underlying TiO_2_ NTs to the EDX analysis, confirmed a 2:1 Cu/O stoichiometry (Supporting Information, Figure S6).

**Figure 2:**
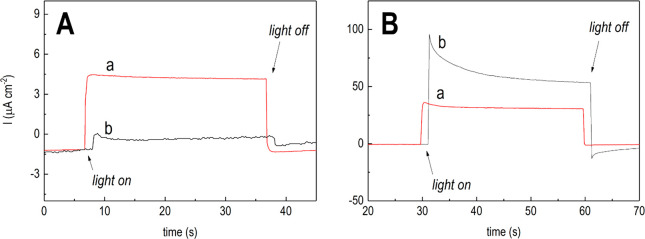
Results from photocurrent tests (AM 1.5G standard) performed under visible irradiation only (A) and UV–vis irradiation (B). Photocurrent transients of the n-TiO_2_/p-Cu_2_O-based electrode (a, red line) and bare n-TiO_2_ (b, black line).

In the latter electrode, able to provide a fair visible light absorption of Cu_2_O,^29,49,50^ the continuous thick Cu_2_O layer (Figure 1D) would produce complete shading of the underlying TiO_2_ NTs, lowering the UV light-harvesting efficiency with respect to bare n-TiO_2_.^41,42^ Accordingly, photocurrent tests were restricted to photoelectrodes produced at 120 mC transferred charge (Figure 2).

In Figure 2, photocurrents generated at zero-bias conditions under only visible light (Figure 2A) or UV–vis light (Figure 2B) are shown for the reported samples. Remarkably, the bare n-TiO_2_ NTs exhibited the largest photocurrent under UV–vis irradiation (Figure 2B, b), corroborating our previous findings.^45^

This unexpected behavior can be explained by considering the distinct sizes of the two semiconductors forming the p–n heterojunction. The photoanode synthesized at 120 mC is mainly characterized by large (∼100 nm) octahedral Cu_2_O NPs, forming heterojunctions on top of the NTs array, where the thickness of the NTs walls is ∼10 nm (Supporting Information, Figure S4). It is well known that the potential distribution and width of the space-charge layer depend on the amount of charges transferred across the p–n interface and hence on the density of the majority charge carriers in the two semiconductors.^5^ Therefore, even if the thickness of the NTs walls would be entirely composed by majority charge carriers (i.e., electrons for n-type), the depth of the resulting space-charge layer in the p-side of the junction could be at most of the same size. It is therefore clear that for 100 nm NPs this corresponds to a low *W*_p_/*L*_p_ ratio (Supporting Information, Figure S2; *W*_p_ ≪ *L*_p_), limiting the useful device thickness. In this way, charge carriers photogenerated too far from the p–n interface cannot contribute to determine the Δ*V*_photo_^29^ responsible for the p–n-based photoanode performance.

In addition to the above results, further space-charge layers at the p-Cu_2_O/electrolyte and n-TiO_2_/electrolyte interfaces form as well (Supporting Information, Figure S1(III) and S1(I), respectively). Following the above considerations, since the p-Cu_2_O NPs are surrounded by the electrolyte, the increased contact surface determines a more efficient interface as compared to the p–n junction discussed above. Considering that Δ*V*_photo_ at the p-Cu_2_O/electrolyte interface opposes the same at the p–n interface, this contributes to determining the overall performance decay of the p–n-based PEC cell. In addition, as the anodic photocurrents invariably attained demonstrate (Figure 2), Δ*V*_photo_ at the p-Cu_2_O/electrolyte interface is outweighed by Δ*V*_photo_ at the p–n and n-TiO_2_/electrolyte interfaces.

Finally, a further explanation for the observed performance decay comes from the absorption characteristics of Cu_2_O, specifically by its optical depth (OD(λ)^29^). In fact, considering that the sizes of the Cu_2_O NPs (∼100 nm) on top of NTs allow it to absorb the UV light almost completely,^29^ the underlying photoactive materials are partly “shaded” by the overlying NPs. Noticeably, also in the absence of such NPs on top of the NTs, the same shading effect could come from the 2 μm thick close-packed NTs array (Supporting Information, Figure S7). Thus, even with p–n heterojunctions preferentially distributed at the bottom of the NTs (Supporting Information, Figure S3B) they cannot contribute efficiently to the overall photoanode operations. Accordingly, it can be assumed that the minimum condition to guarantee an improved photoresponse by p-Cu_2_O electrodeposition would be to form p–n heterojunctions preferentially distributed on top of the NTs array.

Conversely, under only visible light the p–n-based photoanode showed the largest photocurrent (Figure 2A, a). This indicates that the composite electrode invariably follows the p–n-based photoanode operations also when n-TiO_2_ is almost photoinactive (Figure 2A, b). However, such photocurrent remains more than 1 order of magnitude lower as compared to that registered under UV–vis irradiation (Figure 2B, a). The latter evidence demonstrates that the performance achieved under visible light only cannot be considered as improved. To this purpose, we consider that the p-Cu_2_O electrodeposition was used as a tool to extend the light absorption window of the bare n-TiO_2_ photoanode to the visible range. Improved performance can be claimed only after a comparison with the bare n-TiO_2_ substrate under UV–vis irradiation.

**Figure 3:**
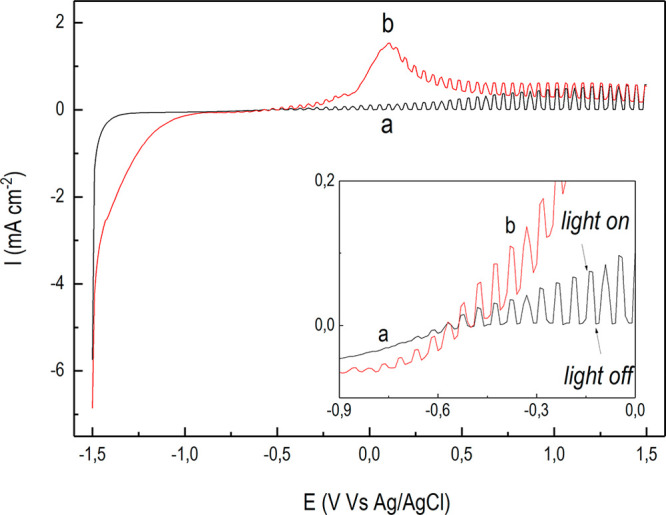
PL-LSV characterization under UV–vis irradiation for: the bare n-TiO_2_ (a, black line) and for the *n-*TiO_2_/*p-*Cu_2_O-based electrode synthesized at 120 mC (b, red line). (Inset) Magnification in the potential range where anodic photocurrents starts to appear.

**Figure 4:**
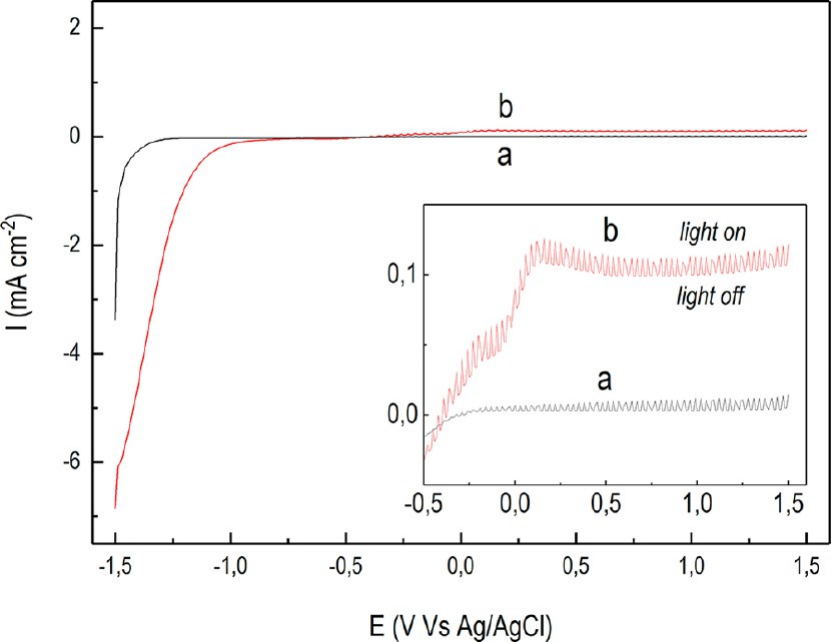
PL-LSV characterization under only visible irradiation (λ > 400 nm) for bare n-TiO_2_ (a, black line) and for the n-TiO_2_/p-Cu_2_O-based electrode synthesized at 120 mC (b, red line). (Inset) Magnification in the potential range where anodic photocurrents starts to appear.

The p–n-based photoanode was further characterized by pulsed light linear sweep voltammetry (PL-LSV), which consists of a classic LSV carried out under chopped light irradiation (Figures 3 and 4). This test can clarify whether direct bias applications can induce improved photoresponse.^48^

Figure 3 shows the PL-LSV characterization under UV–vis irradiation for the bare n-TiO_2_ (a, black line) and for the p–n-based photoanode (b, red line). Over the cathodic range from −1.5 to ∼−0.8 V, the irradiation does not significantly affect the cathodic current and an almost continuous profile was attained with barely distinguishable differences between dark and light periods. This indicates that upon irradiation heterojunctions are operating under reverse biased conditions.^51^ Moving toward more anodic potentials, the additive effect of the irradiation results in a net increase of the anodic current in correspondence of each light pulse. This indicates direct biased conditions for the p–n-based photoanode.

As compared to the bare n-TiO_2_ (Figure 3a), the p–n-based photoelectrode (Figure 3b) showed a larger anodic photocurrent up to ∼1.3 V, eventually approaching it at ∼1.5 V. This would indicate improved photoelectrochemical performance triggered by direct bias applications up to 1.3 V.^39,43^ In the potential range from ∼1.3 to 1.5 V, instead, direct bias applications improving the photon-to-current efficiency^6^ reached a maximum for both electrodes tested.

However, on analyzing the photocurrent profile, the larger anodic photocurrent produced by the p–n-based photoelectrode (wide peak at ∼0,1 V; Figure 3b) is more consistent with oxidation phenomena occurring on the electrodeposited Cu_2_O^48^ rather than with oxidations catalyzed by the same photoelectrode.^39,43^ In fact, as supported by several authors,^41,52,53^ such anodic peak could be attributed to oxidations promoted by the synergistic effect of the photogenerated holes (eqs 1 and 2) with the applied potential
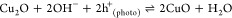
1
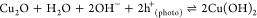
2

To better clarify the relative weight of the two contributions, the same characterization was performed under only visible light (Figure 4), i.e., when the bare n-TiO_2_ is photoinactive. As shown in Figure 4b for the p–n-based photoelectrode, an anodic photocurrent was still generated at sufficiently large potentials, while it became negligible for the bare n-TiO_2_ (Figure 4a). However, although an anodic peak at ∼0.1 V was still present, its intensity decreased by more than 1 order of magnitude. This may emphasize the n-TiO_2_ role in determining the oxidation phenomena described above.

In fact, considering that under visible-only irradiation (Figure 4b) the TiO_2_ photoresponse is inactivated, the largest photocurrent achieved under UV–vis irradiation (Figure 3b) can be attributed to the holes photogenerated in the UV-active TiO_2_, promoting the Cu_2_O photoassisted oxidation (eqs 1 and 2). From this perspective it can be assumed that the Cu_2_O oxidation was mainly determined, in order of contributions, by the holes photogenerated in the TiO_2_, by the applied potential, and/or by the holes photogenerated in the Cu_2_O (visible-only contribution). Referring to the latter, it is noticeable that the occurrence of Cu_2_O “self-degradation” would correspond to a progressively variable photocurrent during each illumination period of the photocurrent test performed at zero bias.^54^ Conversely, in the present study, for the p–n-based electrodes, an almost flat photocurrent profile was attained (Figure 2a). This suggests that under visible-only irradiation direct bias application is required to eventually trigger the photocorrosion of the electrodeposited Cu_2_O.^54^

### Improving the Photoresponse of the Synthesized n-TiO_2_/p-Cu_2_O-Based Photoanodes

The previous characterization study performed highlighted that in order to prevent a performance decay of the nanosized p–n-based photoanode, the optimum condition is to form p–n heterojunctions preferentially distributed on top of the NTs array.

At the same time, efficient visible light harvesting of p-Cu_2_O in the visible region implies an increasing NPs size toward the microscale (at least ∼1 μm^55^), which conversely corresponds to an overall decrease of the photoresponse of the underlying materials. Hence, a major challenge to effectively boost the p–n-based photoanode performance under UV–vis irradiation is to guarantee an increased visible-light-harvesting efficiency for p-Cu_2_O^55^ and an increased efficiency at the p–n interfaces^29^ (Supporting Information, Figure S2, where *W*_p_ ≈ *L*_p_).

**Figure 5:**
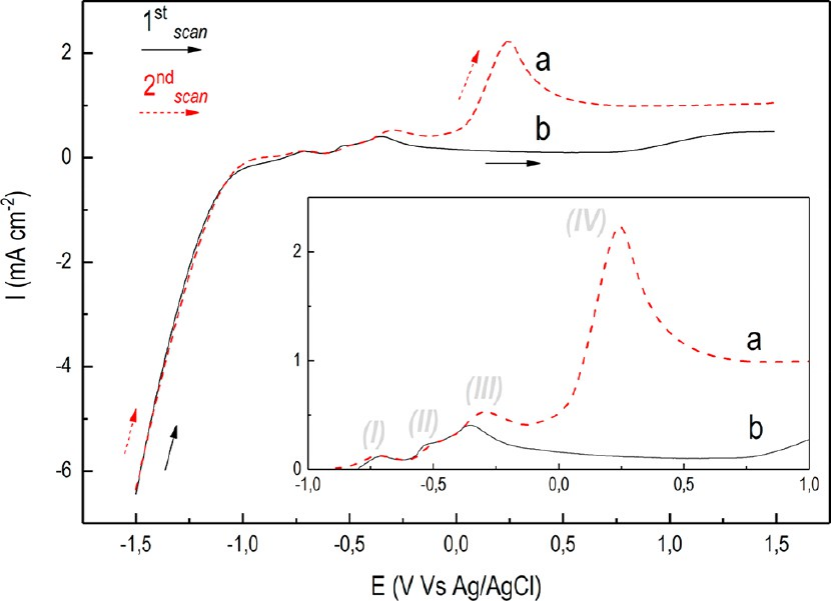
Double-scan LSV-based post-treatment. Current behavior of the n-TiO_2_/p-Cu_2_O-based electrode electrodeposited at 120 mC: first scan in the dark (b, black line), and second scan under UV–vis (AM 1.5G standard) irradiation (a, red dashed line). (Inset) Magnification of the oxidation peaks observed (I–IV).

In the present study, the strategy followed was an electrochemical post-treatment able to induce suitable modifications of the originally electrodeposited p-Cu_2_O. In order to modify the Cu_2_O nanostructures, LSVs on the p–n electrodes were preliminary performed by varying the applied potential from −1.5 to 1.5 V. The underlying idea was to promote an electrochemical modification of the deposited Cu_2_O NPs by application of the cathodic potentials at the early stage of the LSV, thus partly reducing the copper oxide deposits, and then to progressively reoxidize the deposits to restore the p-Cu_2_O photoactive phase. In this scenario, considering the pivotal role of n-TiO_2_ in inducing photo-oxidation phenomena (Figure 3), three different post-treatments of the composite electrodes were evaluated: (i) LSV without illumination (dark LSV), (ii) LSV under simulated sunlight (light LSV), and (iii) the two above treatments in a sequence (double LSV, first scan dark and second light). Figure 5 shows the evolution of the currents generated performing the double LSVs, which qualitatively reproduces the behaviors of the other two treatments when separately investigated (Supporting Information, Figure S8). For all of the treatments tested, the cathodic current at the early scans can be assigned to the water reduction (eq 3) and to a partial reduction of the Cu_2_O to Cu(0) (eq 4)
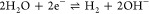
3
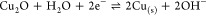
4

During the lighted scans (Figure 5a; Figure S8B in the Supporting Information), the reduction of Cu(I) to Cu(0) can include the reductive decomposition of Cu_2_O (eq 5,^56^) through the p-Cu_2_O/electrolyte interfaces
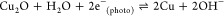
5

Moreover, considering the surface composition of the deposited NPs characterized by XPS analysis (Supporting Information, Figure S9), the reduction of the Cu(II) species (eqs 7–10) must be considered too. In fact, the XPS spectra taken in the Cu 2p energy range 930–965 eV (Figure S9A and S9D) revealed the presence of both Cu(I) (932.5 eV) and Cu(II) species (935 eV, plus the Cu(II) satellite lines in the range 940–950 eV) (Figure S9D). The XPS-excited Auger spectra of the sample fall at a kinetic energy of 1849.7 eV (data not shown), which calls for an assignment to Cu_2_O^57^ and coupled with the XPS results allows one to exclude the presence of Cu(0) within XPS detection limits (0.1 surface atom %). To assign the Cu(II) signals to CuO, Cu(OH)_2_, or both, the O 1s and C 1s regions of the spectra were also analyzed. For the bare NTs-based electrode, the signal at ∼531 eV (Figure S9B) represents the metal oxide contribution due to TiO_2_. For the composite electrodes, the presence of Cu_*x*_O species shifts this component to lower binding energies (∼530 eV), and a second weaker signal due to Cu(OH)_2_ appears at ∼532 eV (Figure S9E). The C 1s region (Figure S9C and S9F) for the bare TiO_2_ electrode is a weak and complex signal, containing C–C and C–O components (Figure S9C). For the p–n electrode (Figure S9F), the major contribution comes from C–C bonds (∼285 eV) followed by C–OH (∼286 eV) and HO–C=O groups (∼288 eV) and a very weak component at ∼289 eV due to carbonate ions. The above findings are in close agreement with the data reported by Zhu et al.^58^ for Cu_2_O deposits obtained along a similar procedure employing organic ligands. The presence of surface carbonate, reported by Zhu et al., is in fact interpreted by these authors as a stabilizer of Cu(OH)_2_. Hence, by a combination of XRD and XPS analyses, the composition of the NPs was addressed. The presence of Cu_2_O crystal phases and the absence of Cu(II) peaks were ascertained by XRD (Supporting Information, Figure S5c), while the XPS composition of the NPs surface layer (Figure S9) gave, in order of abundance, Cu(OH)_2_ and Cu_2_O as the main species.^59,60^ In copper surface deposits exposed to air where Cu(OH)_2_ is present as a top surface layer, the presence of CuO underneath Cu(OH)_2_ cannot be confidently excluded. Moreover, since DRS characterization (Figure 7b) excludes the presence of absorption peaks related to CuO, the NPs composition is consistently assigned to Cu_2_O covered by a surface layer of Cu(OH)_2_/Cu_2_O.

**Figure 6:**
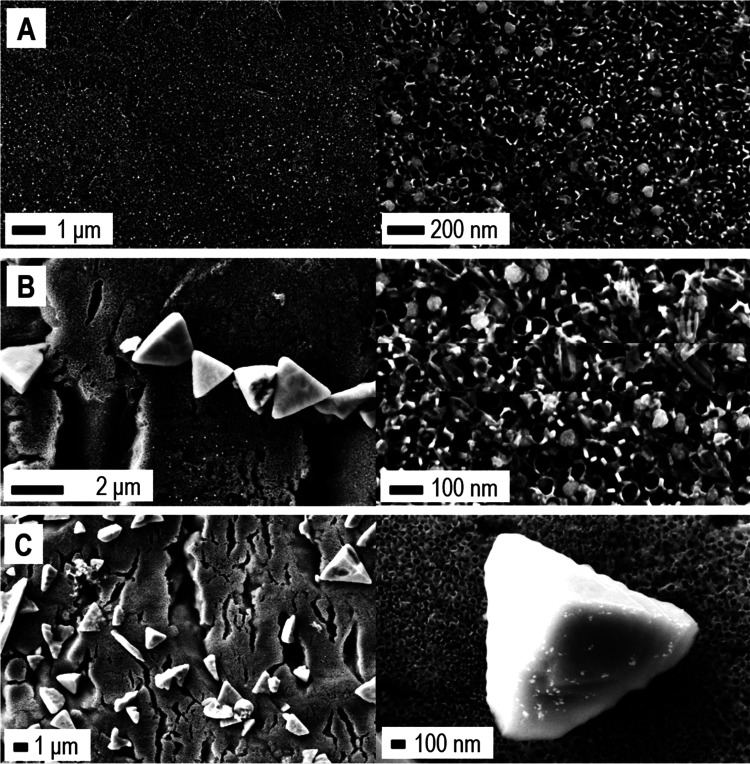
FE-SEM characterization. *n-*TiO_2_/*p-*Cu_2_O electrode electrodeposited at 120 mC: after dark LSV (A), lighted LSV (B), and after the two successive LSVs, first in the dark and second upon illumination (C). Each system is imaged at two levels of magnification, as indicated.

At the early stage of the LSV scan, the reduction of TiO_2_ to Ti cannot be excluded. However, FE-SEM images show (Figure 6) that the NTs array was still present after each condition tested, suggesting that the polarization does not significantly affect the nanotubular architecture of the TiO_2_ NTs substrates. At increasing potentials, three oxidation peaks can be distinguished (Figure 5a and 5b; Figure S8 in the Supporting Information, insets I–III). On the basis of the above discussion, a set of possible reactions that can justify such oxidations are listed below
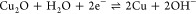
6
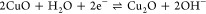
7
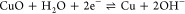
8
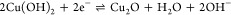
9
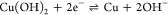
10
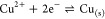
11
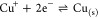
12

A fourth oxidation peak around 0.2 V was only found during the lighted scans (Figure 5a; Figure S8B in the Supporting Information, inset IV). Such oxidations, as for the PL-LSV characterization (Figure 3b), can be due to oxidation phenomena promoted by the n-TiO_2_.

The treated p–n-based photoanodes were further characterized by FE-SEM analysis (Figure 6). After the stand-alone dark scan (Supporting Information, Figure S8A), the original octahedral Cu_2_O NPs covering the top of the NTs (Figure 1C) evolved into rounded particles with lower sizes of ∼50–100 nm (Figure 6A).

After the stand-alone lighted scan (Supporting Information, Figure S8B), new larger tetrahedral clusters (size ≈ 1–2 μm) were formed (Figure 6B). Such a relevant difference suggests that the formation may be due to the photomediated oxidation taking place around 0.2 V (Supporting Information, Figure S8B, inset IV).

**Figure 7:**
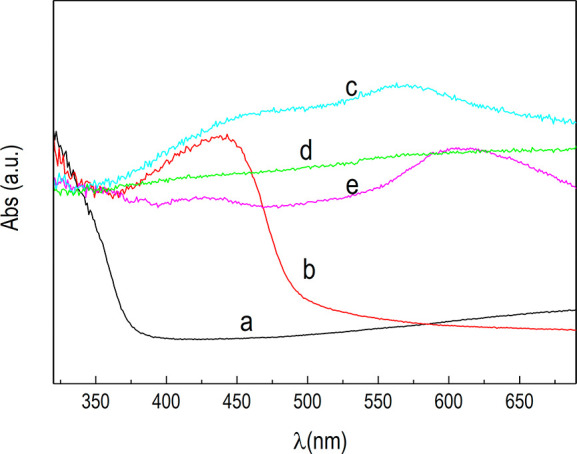
Equivalent absorption spectra. Anatase TiO_2_ electrode (a, black line) and n-TiO_2_/p-Cu_2_O-based electrodes (120 mC): not treated (b, red line), dark LSV treated (c, cyan line), lighted LSV treated (d, green line), and double LSV treated (e, magenta line).

In the case of the electrode produced by the sequence of the two scans (Figure 6C), the FE-SEM images revealed again the presence of the large tetrahedral clusters. In addition, some planar structures comparable in sizes appear (Figure 6C, lower magnification). As reported by several authors, a tetrahedral morphology would be consistent with the formation of CuO,^61,62^ which is characterized by a lower band gap as compared to Cu_2_O.^26^ This is corroborated by the qualitative analysis of the pseudoabsorbance spectra collected at the end of each treatment (Figure 7), where significantly increased absorptions toward higher wavelengths were observed in the visible (Figure 7c–e).

On the basis of the above analysis it can be assumed that the oxidation peak observed only for the treatments performed upon irradiation correspond to the following photo-oxidations mediated by n-TiO_2_
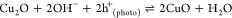
13
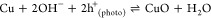
14The photoelectrochemical performance of the LSV-treated electrodes was then evaluated through photocurrent tests at zero bias by measuring the OCP of the PEC cell in the dark, which is then applied during the photocurrent test to guarantee zero current in the dark periods and photoresponse upon irradiation. For all of the LSV-treated electrodes, a progressive variation of the OCP was observed (data shown for the first cycle of the stand-alone dark and light LSV-treated samples in the Supporting Information, Figure S10A and S10B, respectively). These were marked by the onset of cathodic currents during the dark periods, accompanied by the evolution of the (anodic) photocurrent transients during the following irradiation periods.

As stated above, variations of the photocurrents during the irradiation periods are more consistent with phenomena occurring on the Cu_*x*_O deposits rather than with electrochemical reactions catalyzed by the same photoelectrode.^48^ On the other hand, cathodic currents in the dark indicate reverse-bias conditions rather than the zero-bias set at the start of the photocurrent test.

From this perspective it can be assumed that the observed currents are the result of cyclic oxidation/reduction taking place on the same p–n-based photoanode (i.e., photoassisted oxidations during lighted periods followed by reductions in the dark).

On the basis of this assumption, the photocurrent test was optimized to be part of the post-treatment procedure, which was started with the LSVs. For this purpose, the photocurrent tests were carried out by periodically interrupting the experiment (after a fixed number of dark/light intervals, corresponding to one cycle), measuring the “new” OCP of the PEC cell in the dark, and restarting the test (data shown for the dark LSV-treated sample in the Supporting Information, Figure S11(I)). This procedure was repeated until the current generated during the dark periods was no longer appreciably deviating from zero, confirming that the PEC cell was operating at zero bias (Supporting Information, Figure.S11(I); fifth/sixth cycle).

The instability evidenced by the progressive variation of the OCP during this procedure was confirmed by the absorption curves collected at the end of the different cycles (Supporting Information, Figure.S11(II)). In fact, at the end of the LSV treatments the absorption profiles showed increasing intensities toward higher wavelengths in the visible region (Figure 7c–e), indicating the presence of Cu_*x*_O species (mainly CuO) resulting from the oxidations described above (eqs 6–10, 13, and 14). Proceeding with the OCP adjustment procedure, the absorption profiles qualitatively evolved for the cyclic oxidation/reduction phenomena taking place, eventually approaching the original shape of the untreated p–n-based photoelectrode (Supporting Information, Figure S11(II)a and S11(II)d). Specifically, the absorption peak at ∼550 nm that appeared after the dark LSV (Supporting Information, Figure S11(II)b) vanished after the third cycle of the OCP adjustment procedure, while the original peak reappeared at ∼460 nm, accompanied by a second peak at ∼650 nm (Supporting Information, Figure S11(II)c). The latter contribution disappeared when a stable OCP was reached, and the only absorption peak in the visible region remained at 460 nm (Supporting Information, Figure S11(II)d). In this case, the absorption can be exclusively imputed to Cu_2_O.^46^

On the basis of this evidence, it can be assumed that the large tetrahedral CuO deposits achieved as a result of the lighted scans (Figure 6B and 6C) are first reduced to Cu_2_O during the dark periods of the OCP adjustment procedure (data shown for the first cycle of the lighted LSV-treated sample in the Supporting Information, Figure S10B) and then reoxidized to CuO during the lighted periods, cyclically proceeding until formation of the more stable Cu_2_O phase.

For the sample undergoing only a dark LSV scan, it can be assumed that the rounded particles are mainly composed of Cu(II) species and Cu_2_O (Figure 7c, absorption at ∼460 nm). This way, during the OCP adjustment procedure, copper species are oxidized to CuO during the lighted periods and then reduced to the stable Cu_2_O.

**Figure 8:**
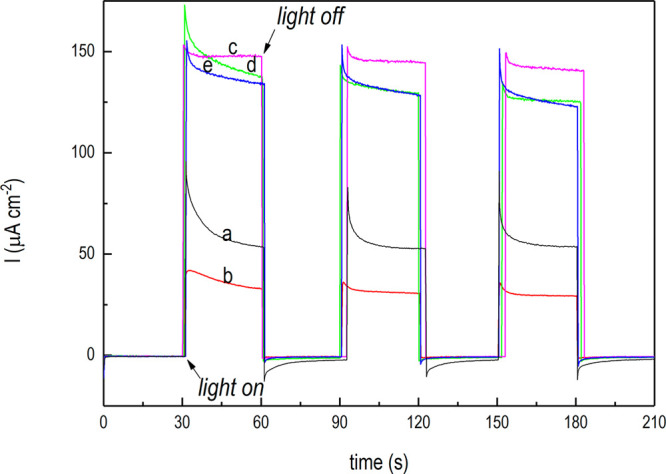
Photocurrent tests under UV–vis irradiation (AM 1.5G standard). Photocurrent transients under chopped light irradiation of the bare TiO_2_ (a, black line) and n-TiO_2_/p-Cu_2_O-based electrodes electrodeposited at 120 mC: untreated (b, red line), dark LSV treated (e, blue line), lighted LSV treated (d, green line), and double LSV treated (c, magenta line). For all of the LSV-treated electrodes, photocurrent transients were measured after the OCP stabilization procedure.

The photocurrent transients generated with the treated electrodes after the OCP adjustment procedure are reported in Figure 8. As shown, photocurrents registered at zero bias (Figure 8c–e) were about three times larger than that attained with the untreated electrode (Figure 8b) or with the bare n-TiO_2_ (Figure 8a), irrespective of the treatment.

**Figure 9:**
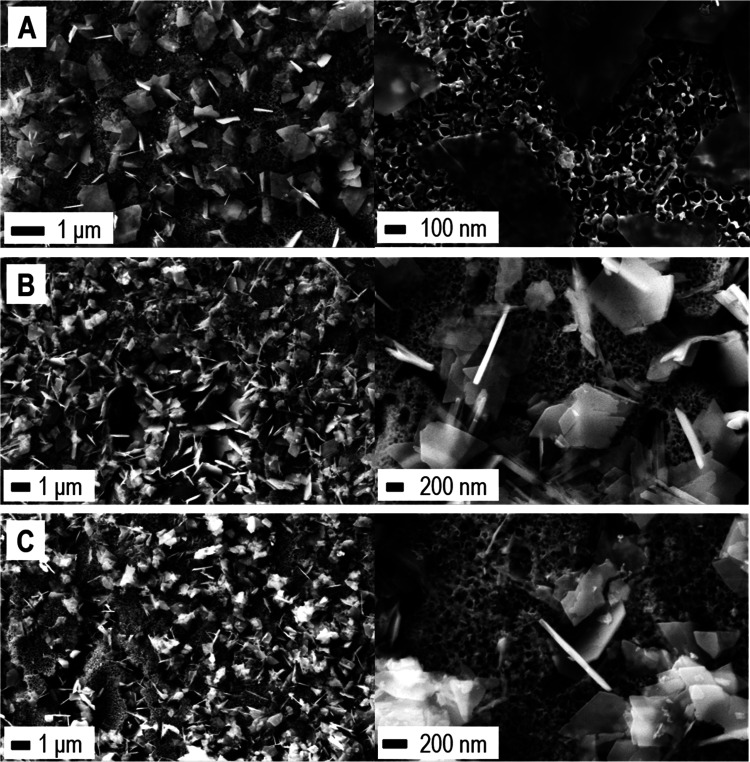
FE-SEM characterization. *n-*TiO_2_/*p-*Cu_2_O-based electrode electrodeposited at 120 mC after the OCP adjustment procedure following (A) dark LSV, (B) lighted LSV, and (C) double LSV (first scan dark, second scan lighted). Each system is imaged at two levels of magnification, as indicated.

Further insights into the mechanisms which determined the improved performance of the treated electrodes can be derived by the FE-SEM images collected at the end of the OCP adjustment procedure, when the electrodes reached the end of their stability (Figure 9). As shown, a network of planar structures characterizes the top of the NTs array. Specifically, the original octahedral NPs (Figure 1C) evolved in 2D leaf-like structures a few tens of nanometers thick and ∼1 μm wide (Figure 9).

The restructuring of the deposits into 2D leaves improved the performance in reason for the reduced Cu_2_O thickness and of the increased contact surface at the p–n interfaces, while the increased width of the 2D structures allowed reaching an improved visible-light-harvesting efficiency for the p-Cu_2_O.^29^ Furthermore, the induced deposits restructuring did not change the preferential distribution of the p-Cu_2_O nanostructures on top of the TiO_2_ NTs array. In other words, since the TiO_2_ NTs walls sizes are the major bottleneck for an adjustment of the *W*_p_/*L*_p_ ratio at the p–n interfaces (Supporting Information, Figure S2), the change of morphology from Cu_2_O NPs (100–150 nm apparent diameter) to 2D leaves (thickness 10–50 nm, width ≈ 1 μm) had two major effects. On one side, it prevented recombination phenomena in the p-side by the optimized *W*_p_/*L*_p_ ratio at the p–n interfaces (Supporting Information, Figure S2; *W*_p_ ≈ *L*_p_), and on the other, it provided the optimal sizes to guarantee improved visible-light-harvesting efficiency for p-Cu_2_O as a function of its optical depth.^29^ Furthermore, the reduced thickness of the 2D leaves mitigated the distances in the charge-carrier diffusion paths within the p-side of the heterojunction, definitively contributing to improving the photocurrent response.

### Analysis of the Mechanisms Governing Formation of the 2D Leaf-Like Structures

As discussed above, large tetrahedral precursors, comparable to the width of the 2D leaves, appeared only under illumination.

A previous study by Wu et al. demonstrated the possibility to form similar leaf-like structures treating p-Cu_2_O photocathodes under either illumination combined with polarization or polarization only.^52^ The formation of the planar structures was justified by considering that irradiation and electrochemical polarization may, in principle, result in partial dissolution of the Cu_2_O, which may later recrystallize at more energetically favorable sites.^52^ However, the same authors clarified that within the restricted potential range investigated, the restructuring of the deposits achieved would be more consistent with the occurrence of atomic surface diffusion rather than with dissolution/redeposition mechanisms.^52^

To clarify these points, in the present study repeated CVs in the dark were separately investigated up to 70 cycles under (i) the same potential window of the LSVs treatments (Supporting Information, Figure S13A) and (ii) in a restricted potential window where the deposits dissolution would be unlikely (Supporting Information, Figure S12A). In this latter range, no anodic peak was evident over the CV cycles, but only a slight increase in the anodic current was observed. The main effect induced on the morphology of the deposits was the transition from the original deposits (Figure 1C) into rounded irregular particles characterized by increased dimensions (Supporting Information, Figure S12B). However, no evidence for the formation of leaf-like structures or large tetrahedral precursors was revealed. In the extended potential range, again, no evidence of the transition to 2D leaf-like structures was evidenced (Supporting Information, Figure S13B–D). The main effect of the repeated CVs was the rearrangement of the original deposits toward eroded NPs (20 cycles), evolving in disordered agglomerates (40 cycles) and then in thicker agglomerates, consisting of disordered nanorods (70 cycles).

By excluding a dissolution/redeposition mechanism for the 2D leaves formation, a photoinduced solid-state transformation^63^ could be assumed. This would be in agreement with the restructuring of the deposits via atomic surface diffusion proposed by Wu et al.^52^ In this scenario, further support could come from charge-transfer dynamics occurring upon irradiation in hybrid plasmonic/semiconductor systems based on wide-band-gap semiconductors (i.e., UV active). In such systems, the strong interaction among the metal nanostructures and the light triggers localized surface plasmon resonance (LSPR) phenomena, which are accompanied by the ultrafast charge transfer from the plasmonic material to the semiconductor.^64^ Accordingly, Pelli Cresi et al.^65^ recently reported on the ultrafast change (<200 fs) in the absorption edge of the semiconductor oxide induced by electrons injection from the excited metal nanostructures.^65^ The plasmonic/semiconductor system investigated was based on an ultrathin semiconductor oxide (comparable to the sizes of the TiO_2_ NTs walls in the present work) which contributed to maximize the LSPR phenomena induced by the interaction among metal and light. In the present study, if we assume a partial reduction of the Cu_2_O deposits to Cu during the post-treatments (e.g., at the early stage of the lighted LSV), the successive photomediated oxidation could have been enhanced by LSPR phenomena occurring in these evolving systems. On this basis, the changes observed at the end of the lighted LSV (i.e., from Cu_2_O NPs to large CuO clusters) could be assigned to the ultrafast photomediated oxidations,^64,65^ while the retrieval of the original composition toward 2D leaf-like Cu_2_O would be determined by recrystallization via atomic surface diffusion, consistent with the findings by Wu et al.^52^

Referring to the plasmon resonance effect, several studies have already reported enhanced TiO_2_ photoactivity.^66−68^ Such kind of resonance phenomena come from the interaction among visible light and metal nanostructures,^66−68^ so that the irradiated plasmon acts as an ”electron pump” for the neighboring semiconductor, enhancing its activity. In the present study, instead, we rather emphasize that the occurrence of resonance phenomena promotes the photoinduced restructuring mediated by the wide-band-gap semiconductor oxide when triggered by polarization (i.e., LSV upon UV–vis irradiation). For this purpose, let us consider the PL-LSV characterization performed in the present study on the p–n-based photoanodes, which was performed under visible (Figure 4b) and UV–vis irradiation (Figure 3b). Since the resonance effect is due to the visible light interacting with the metal nanostructures, any difference in the photoinduced oxidation triggered by the polarization would have been revealed (i.e., same order of magnitude for the oxidation peak at ∼0.2 V) by comparing the two irradiation sources. Conversely, in the present study, the photoinduced oxidation observed under UV–vis irradiation (Figure 3b) was more than 1 order of magnitude higher than the same oxidation under visible irradiation only (Figure 4b), confirming the pivotal role of the UV-active TiO_2_. In addition, in order to evaluate the UV contribution alone to the photocurrent triggered by the polarization, the bare TiO_2_ (Figure 3a) showed, at ∼0.2 V, a photoresponse 1 order of magnitude lower than that observed for the p–n evolving system (Figure 3b). These differences were at the basis of the speculation on the resonance effect discussed above, where the “electron pump” would have enhanced the pivotal role of the TiO_2_ in promoting the photomediated oxidations triggered by the polarization.

## Conclusions

A study on the photoelectrochemical performance of n-TiO_2_/p-Cu_2_O photoanodes was performed, separately investigating the photoactivity under UV–vis and visible-only irradiation. The purpose is to extend the light absorption window for bare n-TiO_2_ toward the visible range by coupling n-TiO_2_ with a p-type semiconductor with a narrower band gap.

By testing the synthesized photoanodes in PEC cells operating at zero-bias conditions, it was demonstrated that the p–n photoelectrodes can effectively work as photoanodes under both UV–vis and visible-only irradiation. Under visible irradiation only, the p–n photoanode exhibited the largest photocurrent, but under UV–vis irradiation, it was outweighed by the bare n-TiO_2_ photoresponse. This trend highlighted that the performance achieved by the p–n-based photoanode under visible irradiation only cannot be considered as improved without performing a comparison with the bare n-TiO_2_ under UV–vis irradiation. In this frame, the mechanisms responsible for the unexpected performance under UV–vis irradiation were analyzed and thoroughly discussed. It was remarked that by employing n-TiO_2_ in the form of NTs, the reduced contact surface at the p–n interfaces strongly influenced the p–n photoanode performance. At the same time, this effect was determined also by the absorption characteristics of the p-Cu_2_O NPs.

In this perspective, a light-induced restructuring of the electrodeposited p-Cu_2_O was performed. Specifically, the application of a photoelectrochemical post-treatment induced the transition of the originally electrodeposited Cu_2_O NPs (3D) into 2D leaf-like structures. Such structures, characterized by a reduced thickness (∼10 nm) and an increased exposed surface (∼1 μm), produced more efficient p–n interfaces and an overall increase in the visible light harvesting. As compared to the bare n-TiO_2_ under UV–vis light, improved performance was definitively attained.

Insights into the mechanisms responsible for the transition toward the 2D leaves revealed the key role played by the irradiation, specifically by n-TiO_2_ exposure under UV–vis light.

## Experimental Section

### TiO_2_ NTs-Based Electrodes Synthesis

TiO_2_ NTs-based electrodes were synthesized through a facile single-step anodization method developed and optimized in our previous works.^46,69^ Briefly, Ti sheets (Alfa Aesar 99.5%, annealed, thickness 0.25 mm) were employed, both as anode and as cathode, in a two-electrode jacketed cell kept at room temperature and magnetically stirred, connected to a power supply (Aim-TTi CPX200DP DC Power Supply Dual Outputs, 2 × 60 V/10 A 180 W). The electrolyte was ethylene glycol (Alfa Aesar, 99%) based, containing 0.3% wt NH_4_F (Alfa Aesar, 98% min.) and 6.0% v/v H_2_O. Single-step anodizations were carried out imposing a cell voltage equal to 60 V, reached at 0.05 V s^–1^, for 27 min. At the end of the anodization crystallization of the amorphous TiO_2_ was enforced by treatment in a muffle furnace (Nabertherm B410, *T*_max_ 1100 °C, 1.2 KW) at 580 °C (heating rate 8 °C min^–1^) for 132.5 min.

The produced anatase TiO_2_-based electrodes were employed as working electrodes for the Cu_2_O electrodeposition, which was performed following a pulsed electrodeposition (PED) method. As summarized in Table 1, this method consists of the cyclic application of a first cathodic pulse (A period) followed by a zero-current time (B period). The duration of any electrodeposition was indirectly fixed in terms of amount of transferred charge (*Q*). Electrodeposition experiments were carried out in a three-electrode cell connected to an IVIUMnSTAT potentiostat with a Pt mesh as the counter electrode and Hg/HgO as the reference electrode, kept at room temperature and magnetically stirred. In order to allow for a swift comparison with results reported in our previous studies,^45,46^ the electrodeposition potential reported hereafter was referred to the Ag/AgCl saturated electrode. CuSO_4_·5H_2_O (Sigma-Aldrich, ≥98%) reagent was employed to prepare the electrolyte solution with a Cu^2+^ content equal to 0.4 M in 3.0 M lactic acid (Alfa Aesar, ACS 85.0–90.0% aqueous solution). The pH of the solution was successively adjusted to 11.0 by addition of NaOH 5.0 M (Merck, ≥99.98%).

**Table 1:** Electrodeposition Methods Tested^a^

	A period		B period		
method	*E*_A_^b^ [V]	*t*_on_ [s]	*I*_B_ [mA]	*t*_off_ [s]	*Q* [mC]
PED1–120	–0.6	0.5	0	5	120
PED1–500	–0.6	0.5	0	5	500

a *E*_A_ is the applied potential during the A period (*t*_on_), *I*_B_ is the current during the B period (*t*_off_), while *Q* is the amount of transferred charge.

b Referred to the Ag/AgCl saturated reference electrode.

### Electrode Characterization

The size and morphology of the TiO_2_ nanotubes and of the electrodeposited Cu_2_O nanostructures were characterized by a field-emission scanning electron microscope (FE-SEM Zeiss Auriga) equipped with an energy-dispersive X-ray analyzer (EDX Bruker QUANTAX 123 eV), employed for elemental analysis of the TiO_2_/Cu_2_O-based electrodes. The electron interaction depth of the punctual EDX analysis performed was 0.2 μm.

The phase composition of the synthesized electrodes was characterized through a X-ray diffractometer (Bruker D8 ADVANCE) with a molybdenum anode (Kα1 = 0.709319 Å), investigating the 2θ range between 10° and 45°. The experimental peak assignments were given by comparison with Crystallography Open Database references,^70^ specifically referring to the following database patterns: [00-901-6190] for Ti, [00-152-6931] for anatase TiO_2_, and [96-100-0064] for Cu_2_O.

The surface atomic composition of the obtained samples was analyzed by X-ray photoelectron spectroscopy (XPS), using a modified Omicron NanoTechnology MXPS system. Experimental spectra were theoretically reconstructed by fitting the peaks to symmetric Voigt functions and the background to a Shirley or a linear function. XPS atomic ratios (∼10% associated error) among relevant core lines were estimated from experimentally determined area ratios corrected for the corresponding theoretical cross sections and for a square root dependence of the photoelectrons kinetic energies. All of the samples were mounted on nonmagnetic stainless steel tips with conductive adhesive tape. On the basis of the surface sensitivity of the XPS analytical technique (2–5 nm), any result coming from XPS application can be considered to come from a <5 nm layer, measured from the surface top.

The optical properties of the semiconductor-based electrodes were investigated through diffuse reflectance spectroscopy with a Shimadzu UV-2600 in the wavelength range 320–700 nm. The resulting reflectance curves were transformed in equivalent pseudoabsorption spectra with the Kubelka–Munk function.^46^

### Photoelectrochemical Characterization

The photoelectrochemical performance of the synthesized electrodes was assessed by the application in a three-electrode jacketed cell (24.5 ± 0.05 °C) using a SOLARTRON 1287 potentiostat. The prepared electrodes were employed as working electrodes, while a Pt spiral wire and saturated Ag/AgCl were employed as the counter and reference electrodes, respectively. The electrolytic solution was 0.01 M Na_2_SO_4_ (Merck, ACS reagent, ≥99.0%).

In the photocurrent tests, the simulated sunlight irradiation was generated by a solar simulator (Asahi Spectra HAL-320, 300 W Xe lamp, AM1.5G solar simulation filter). The tests carried out under only visible light irradiation were performed using the same solar simulator equipped with an UV cut-off filter (λ < 400 nm cutoff wavelengths). An incident light power density of 100 mW cm^–2^, characterized through a luxmeter (Gossen Mavolux digital), was attained, irrespective of the wavelength emission range, in all of the performed tests as well hereafter described.

The photocurrent tests were carried out by keeping the PEC cell at zero-bias conditions, cyclically alternating light to dark for fixed time intervals (30 s). For this purpose, in each test, the open-circuit potential (OCP) of the PEC cell in dark conditions (i.e., zero bias) was initially measured and then applied to the cell prior to providing the illumination cycles. By applying such potential to the PEC cell during the photocurrent test, a zero-current response in the dark periods is attained, alternated to photocurrent generation under irradiation.

A separate photoelectrochemical characterization of the prepared photoelectrodes was performed through the linear sweep voltammetry technique under pulsed light irradiation (PL-LSV), investigating the potential range between −1.5 and 1.5 V, at a scan rate of 20 mV s^–1^, in 0.01 M Na_2_SO_4_ (Merck, ACS reagent, ≥99.0%) electrolyte. The PEC cell employed for this test was the same as that adopted for the photocurrent tests, but an incident light density of 100 mW cm^–2^ was guaranteed by a commercial low-cost sunlight-simulation lamp (OSRAM Ultra Vitalux 300 W), and the circuit was closed by the IVIUMnStat potentiostat. The tests carried out under only visible light were performed again with an UV cut-off filter. The dark to light cycles were guaranteed by a mechanical shutter placed between the PEC cell and the light source. The shutter was connected to the digital output of the potentiostat to control the open/closure frequency during the LSV scans, which was set at 0.66 Hz.

## Supporting Information Available

Different configurations in PEC cells employing semiconductor-based photoelectrodes, schematic representation of p–n interfaces, FE-SEM characterization, XRD characterization, FE-SEM/EDX characterization, pseudoabsorbance and optical depth of the TiO_2_ NTs-based substrate, LSV -based post-treatments, XPS characterization, photocurrent transients of the OCP adjustment procedure and pseudoabsorbance spectra, repeated CVs experiments (PDF)
