# Supplementary material for: Two-Dimensional Restructuring of Cu2O Can Improve the Performance of Nanosized n-TiO2/p-Cu2O Photoelectrodes under UV–Visible Light
Source: ACS Appl Mater Interfaces. 2021 Oct 4;13(40):47932–44. doi: 10.1021/acsami.1c13399 (PMC8517957; doi:10.1021/acsami.1c13399)
Supplement: Supplementary file 1 — am1c13399_si_001.pdf [file am1c13399_si_001.pdf]

Supporting Information

Two-Dimensional Restructuring of Cu_2O can improve the performance of nanosized n-TiO_2/p-Cu_2O photoelectrodes under UV-visible light

Antonio Rubino, Robertino Zanoni, Pier G. Schiavi, Alessandro Latini, Francesca Pagnanelli*

Department of Chemistry, Sapienza University of Rome, P.le Aldo Moro 5, 00185, Rome, Italy

**corresponding author: antonio.rubino@uniroma1.it*

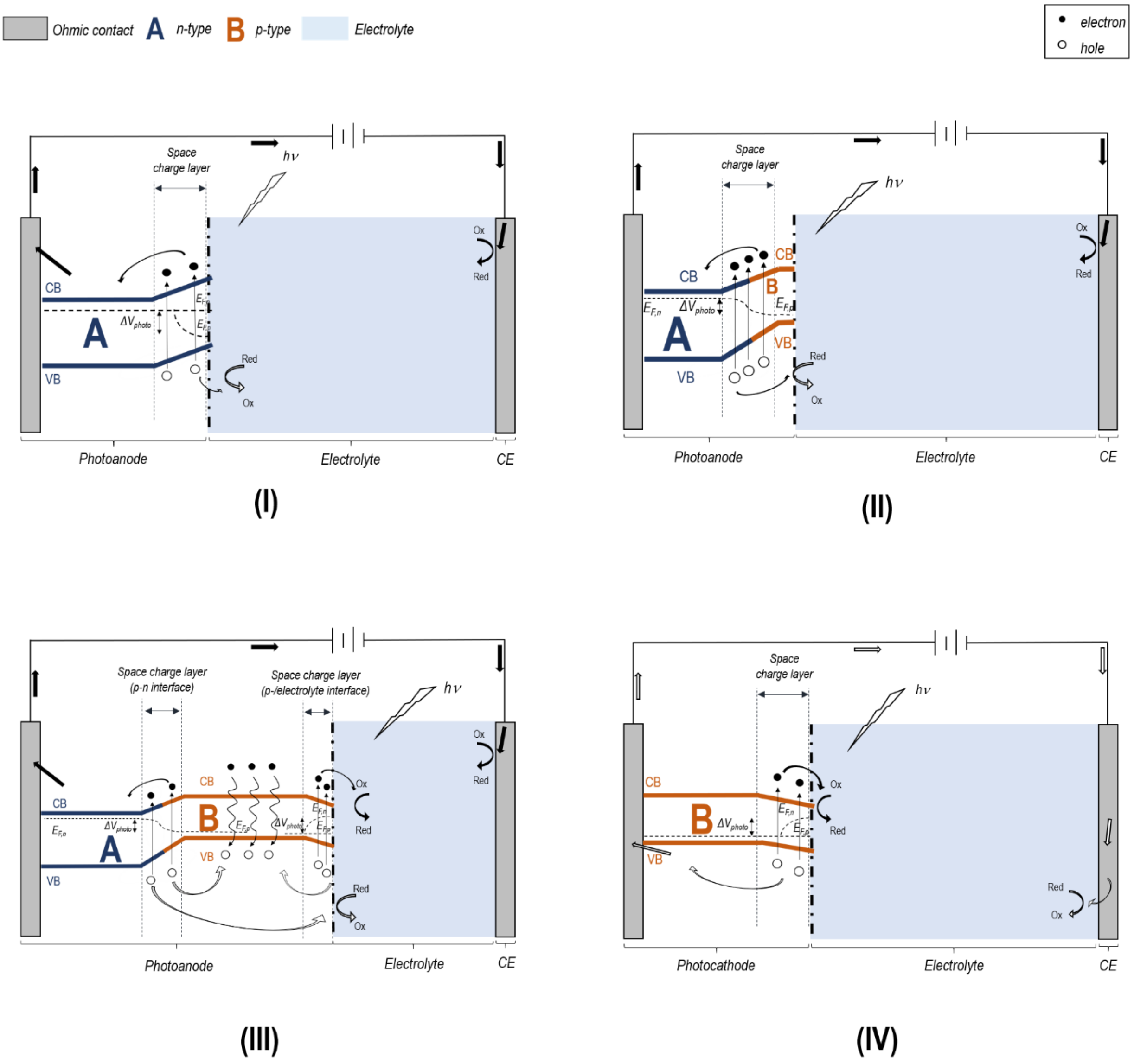

Figure S1. Examples of PEC cell configurations employing semiconductor based photoelectrodes: (I) n-type photoanode; (II) p-n photoanode ($W_p \approx L_p$); (III) p-n photoanode ($W_p << L_p$), including the independent p-type/electrolyte interface formation opposing the p-n photoanode operations; (IV) p-type photocathode. W_p and L_p follow the schematic representation in Figure S2.

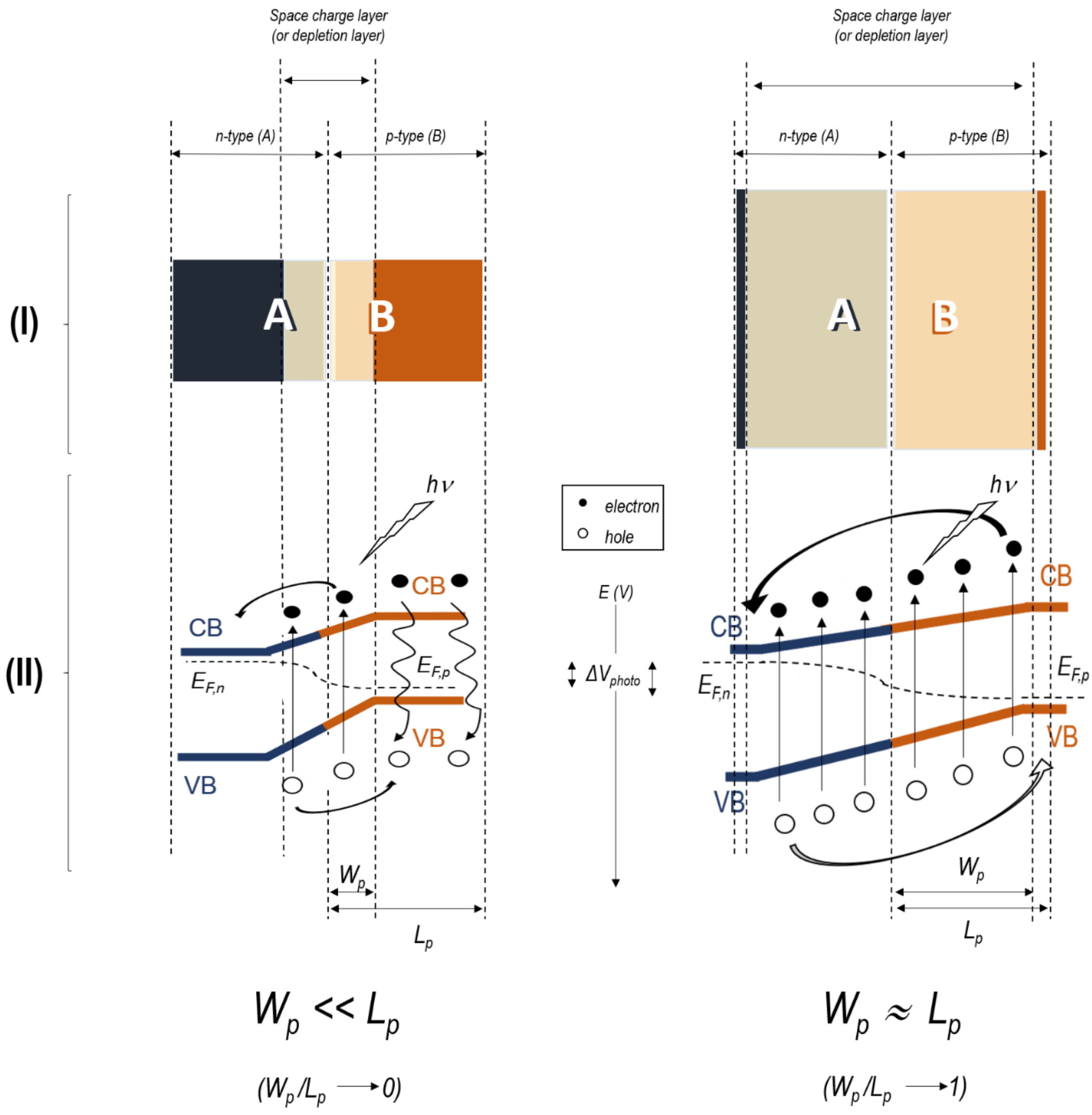

Figure S2. Schematic (2D) representations of p-n interfaces (I) and corresponding energy band models upon irradiation (II): assuming on the left reduced contact surface as compared to the right. CB and VB are the conduction and valence bands respectively. Referring to the p-side, W_p is the width of space charge layer at the p-n interface, while L_p is the width of the semiconductor.

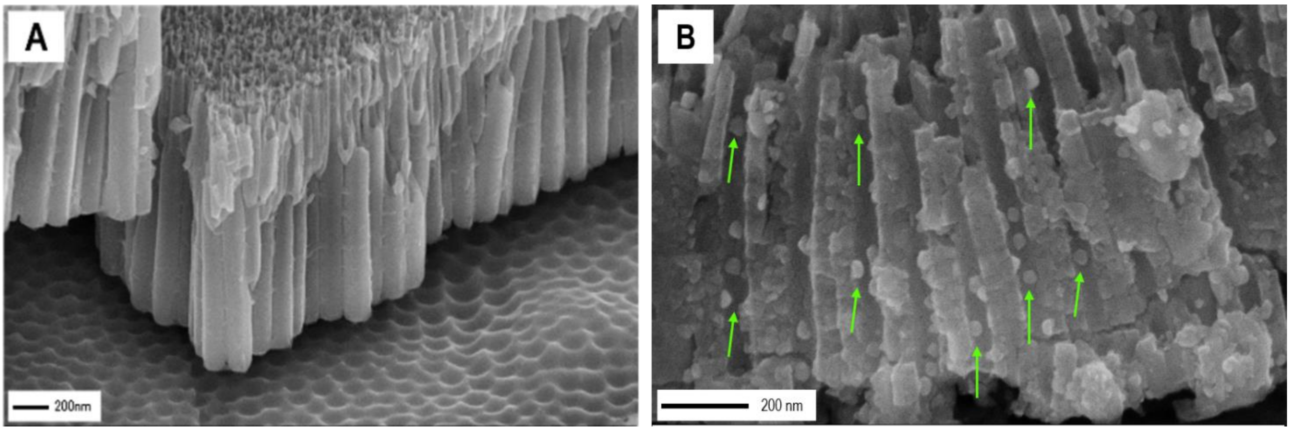

Figure S3. FE-SEM images. Cross sectional view of the bare n-TiO_2 (A) and composite n-TiO_2/p-Cu_2O based electrodes at 120 mC transferred charge. The green arrows indicate the Cu_2O deposits along the TiO_2 NTs walls.

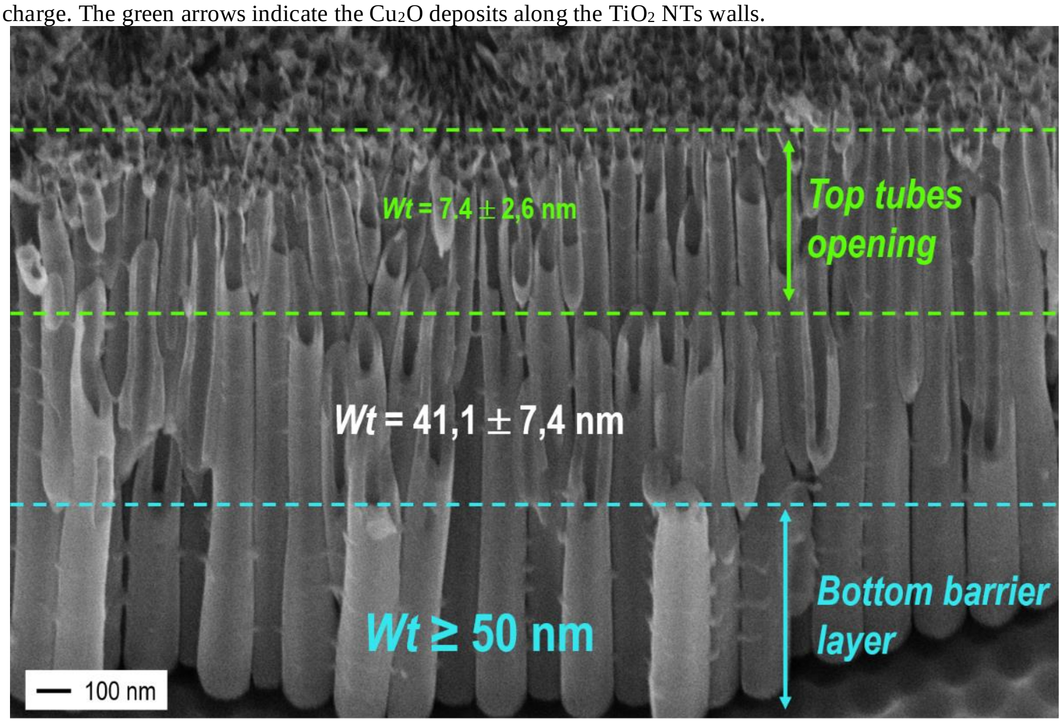

Figure S4. FE-SEM image. Cross section of the TiO_2 NTs, highlighting the different tube walls thicknesses (*Wt*) along the NTs height profile.

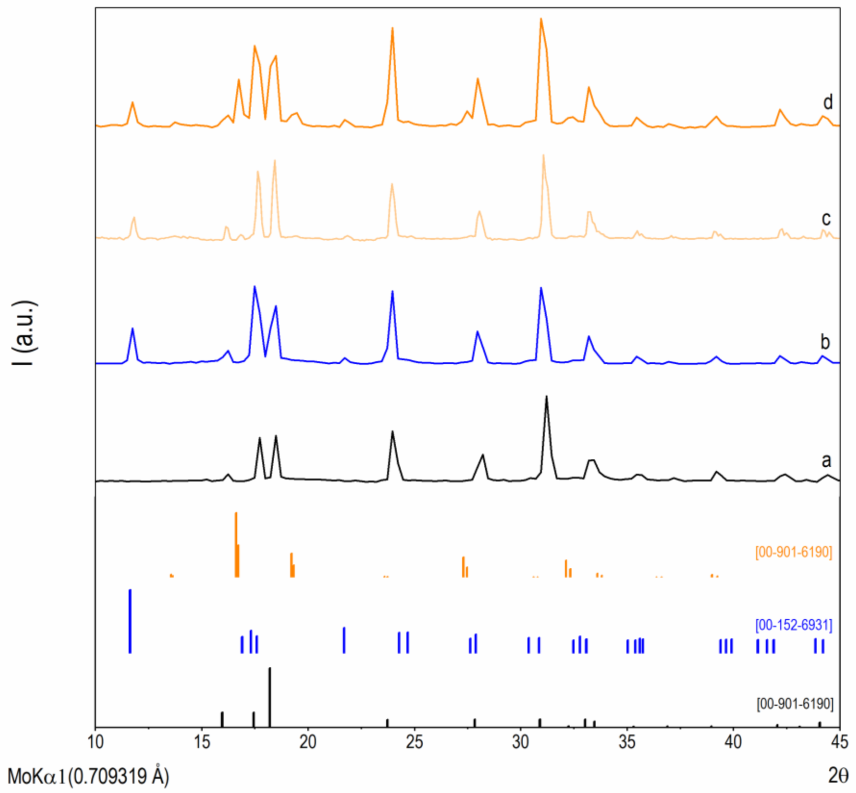

Figure S5. XRD characterization. The bars at the bottom are crystallographic database reference patterns representative of Ti ([00-901-6190], black bars), Anatase TiO_2 ([00-152-6931], blue bars) and Cu_2O ([96-100-0064], orange bars), while the experimental patterns on the top are representative of TiO_2 based electrode as anodized (a, black line), after the thermal treatment (b, blue line) and after the Cu_2O electrodeposition at 120 mC (c, light orange) and 500 mC (d, orange).

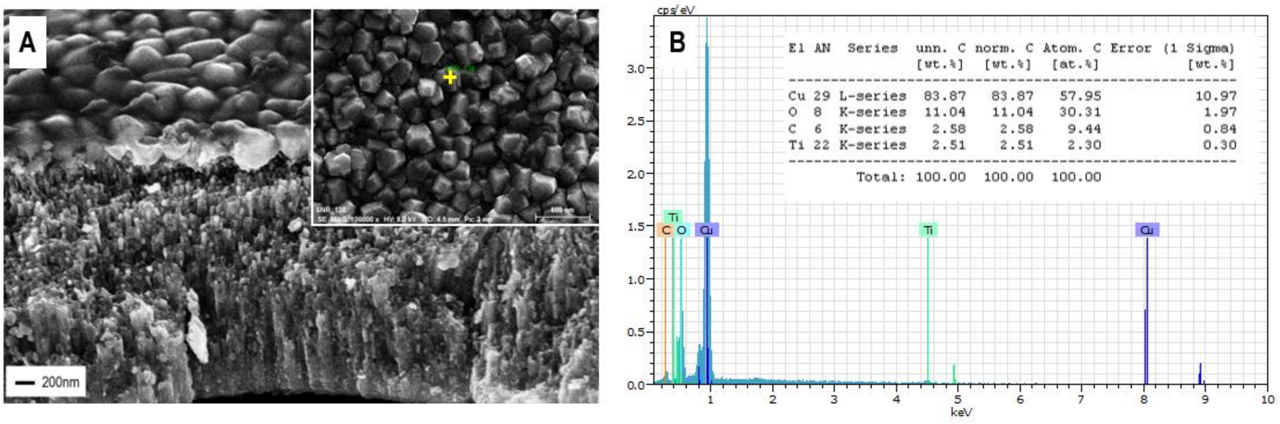

Figure S6. FE-SEM/EDX characterization. A) Cross sectional view of the Cu_2O based electrode synthesized at 500 mC: the yellow cross (top view insert) highlights the EDX punctual analysis. B) EDX spectrum with overlapped the atomic concentrations table (Atom. C column) of the detected elements. The continuous sharped layer (~ 400 nm thickness) of such sample allowed to exclude the underlying TiO_2 contribution to the EDX analysis.

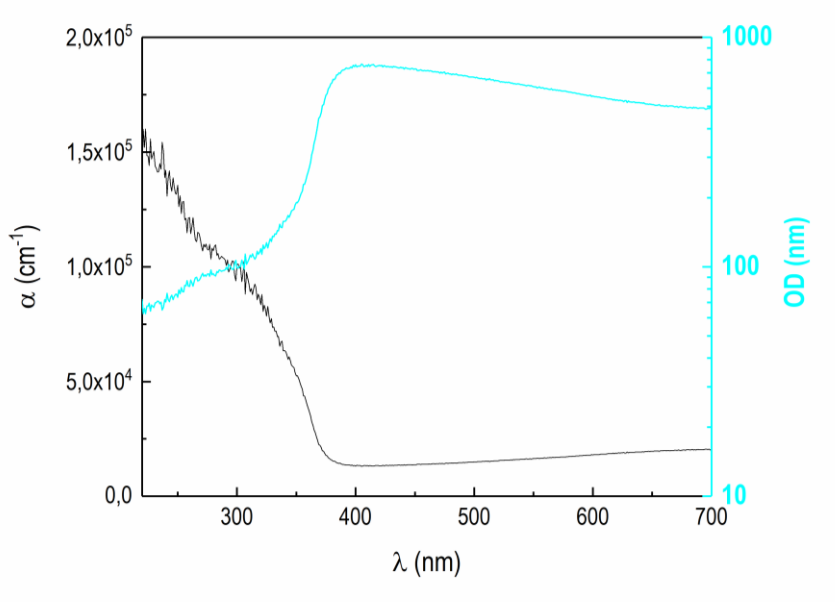

Figure S7. Absorption coefficient (α) and optical depth (OD) for the bare TiO_2 based electrode estimated starting from DRS measurement, assuming an average thickness of 1.8 µm for the TiO_2 NTs array and a negligible contribution of the Ti substrate.

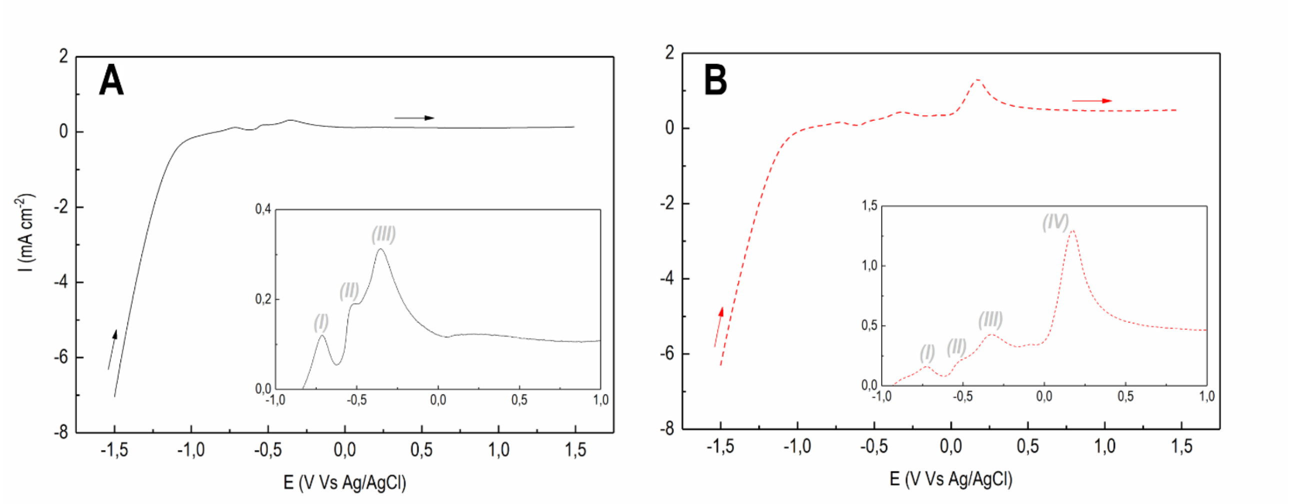

Figure S8. Single scan LSV based post-treatments. Current behaviours for the n-TiO_2/p-Cu_2O based electrode synthesized at 120 mC: (A) dark LSV, and (B) light LSV (AM 1.5G standard). Insets are magnification of the oxidation peaks observed (I-IV).

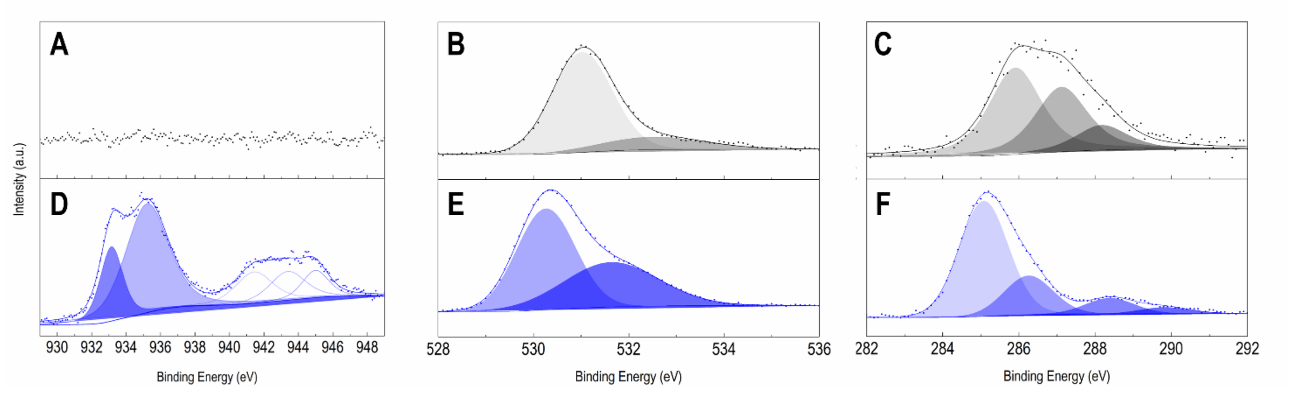

Figure S9. XPS spectra of the bare TiO_2 (A-C) and composite n-TiO_2/p-Cu_2O based electrodes (D-F): Cu 2p region (A and D); O 1s region (B and E) and C 1s region (C and F).

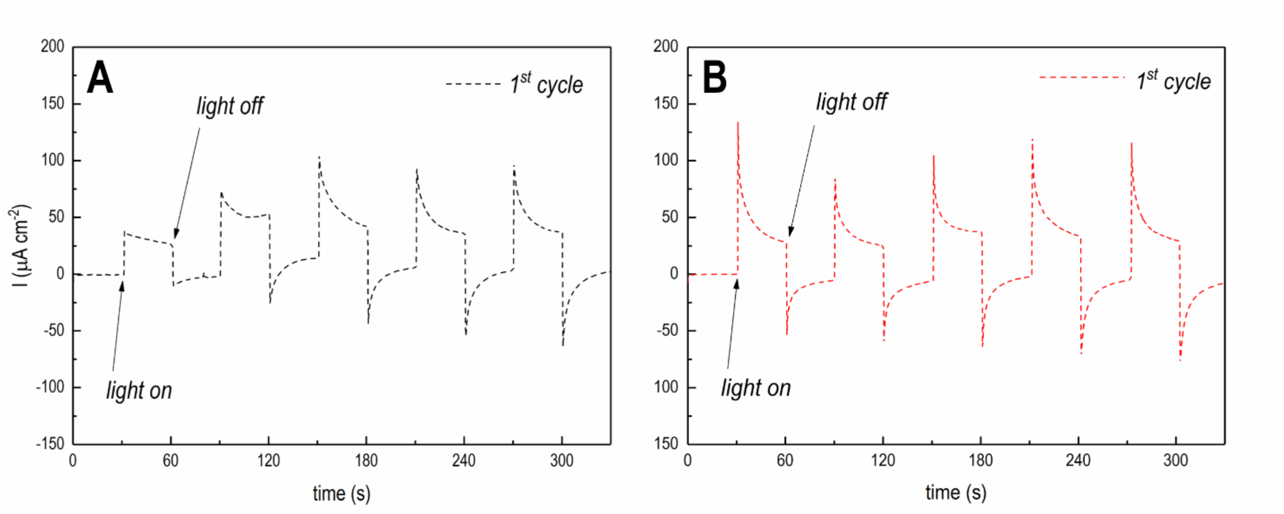

Figure S10. Photocurrent tests (AM 1.5G standard) performed at the end of the LSVs based treatment: (A) 1st cycle after dark LSV and (B) 1st cycle after lighted LSV

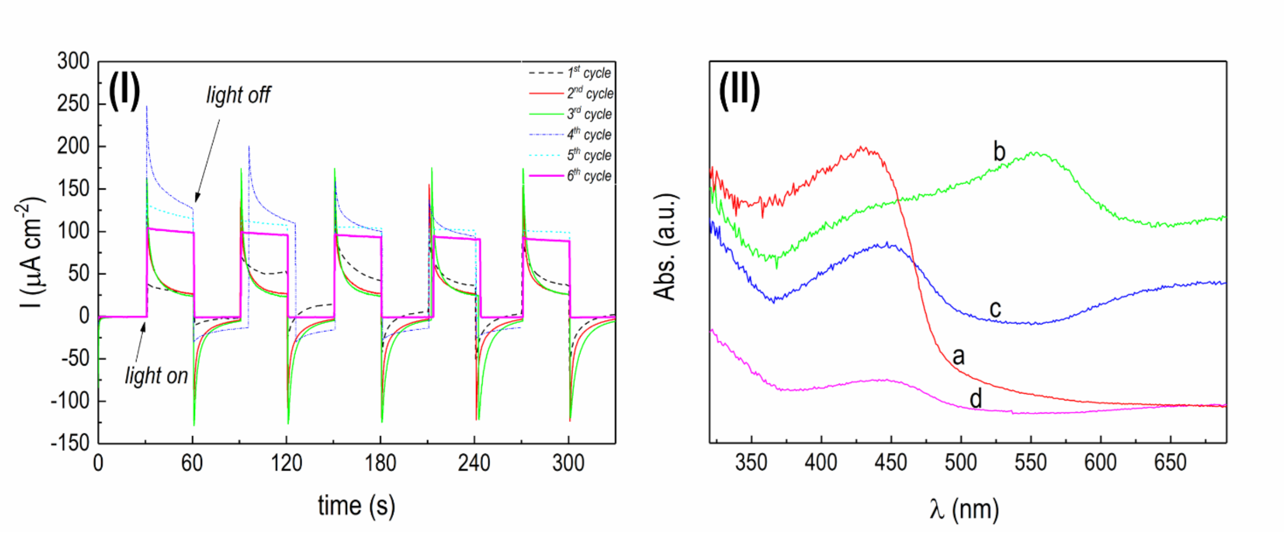

Figure S11. Photocurrent transients registered under chopped light (AM 1.5G standard) during the OCP adjustment procedure after dark LSV treatment (I). Equivalent absorption spectra related to the OCP adjustment procedure (II) for: *n*-TiO_2/*p*-Cu_2O based electrode (120 mC): as electrodeposited (a, red line); after dark LSV (b, green line); dark LSV treated after 3rd (c, blue line) and 5th (d, magenta line) cycles of the OCP adjustment procedure.

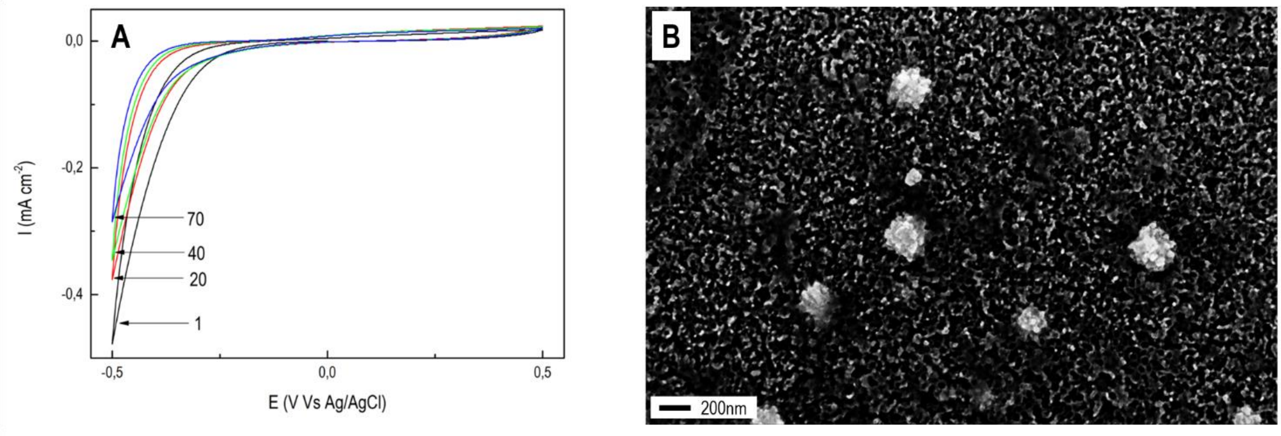

Figure S12. A) Repeated CVs (70 cycles) employing n-TiO_2/p-Cu_2O based electrode (120 mC). B) FE-SEM image of the treated sample after 70 cycles.

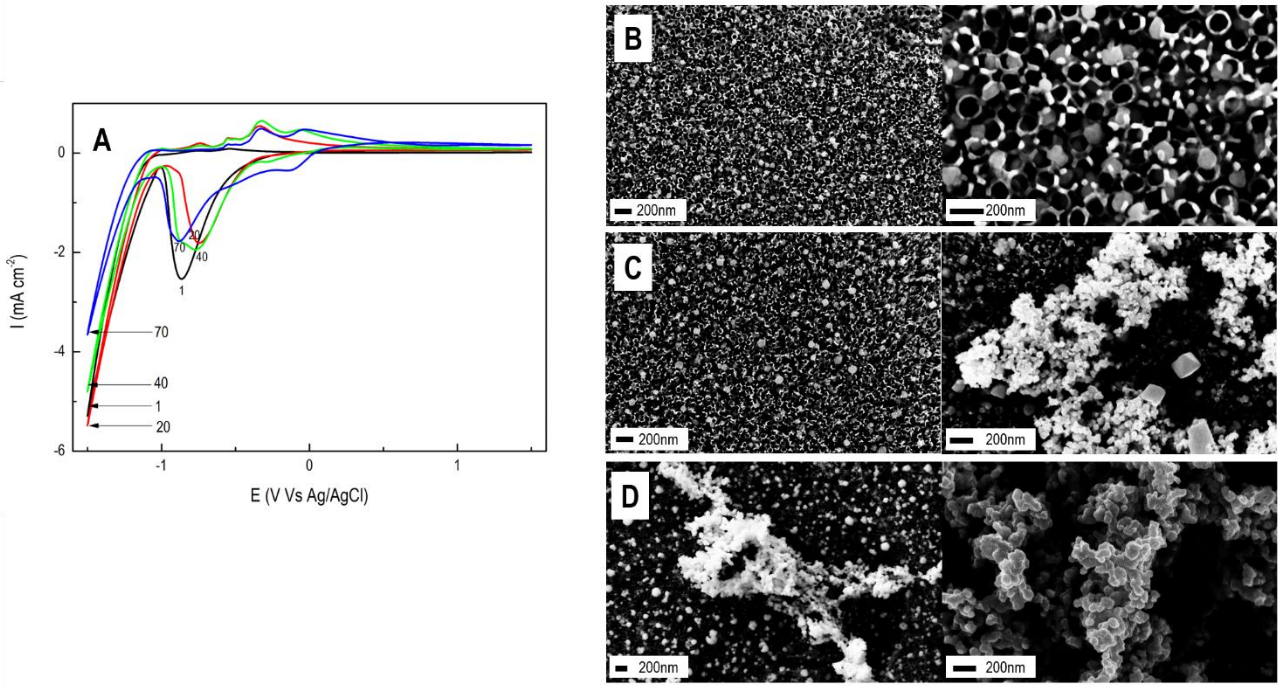

Figure S13. A) Repeated CVs (70 cycles) employing *n*-TiO_2/*p*-Cu_2O based electrode (120 mC) in the same extended potential range investigated during the dark LSV based treatment. FE-SEM images of the samples treated through repeated CVs in the same extended potential range investigated during the dark LSV based treatment: B) 20 cycles; C) 40 cycles and D) 70 cycles. Each condition is represented at two levels of magnification.
